# A Full Computerized Workflow for Planning Surgically Assisted Rapid Palatal Expansion and Orthognathic Surgery in a Skeletal Class III Patient

**DOI:** 10.1155/2022/6413898

**Published:** 2022-10-19

**Authors:** Orazio Bennici, Alessia Malgioglio, Serena Moschitto, Gianrico Spagnuolo, Alberta Greco Lucchina, Vincenzo Ronsivalle, Gaetano Isola, Antonino Lo Giudice

**Affiliations:** ^1^Private Practice, Catania 95123, Italy; ^2^Department of Medical-Surgical Specialties, School of Dentistry, University of Catania, Policlinico Universitario “G. Rodolico-San Marco”, Via Santa Sofia 78, Catania 95123, Italy; ^3^Department of Neurosciences, Reproductive and Odontostomatological Sciences, University of Naples “Federico II”, Naples, Italy; ^4^Regenerative Medicine Research Laboratory, Saint Camillus University of Health Sciences, Rome, Italy

## Abstract

In the present case report, we present and discuss the digital workflow involved in the orthodontic/orthognathic combined treatment of a skeletal malocclusion correction in a 17-year-old male patient affected by a skeletal class III, facial asymmetry, sagittal and transversal deficiency of the medium third of the skull, dental crowding, and bilateral cross-bite. The first stage of the treatment involved surgically assisted rapid palatal expansion and occlusal decompensation, using fixed self-ligating appliance. An orthodontic software package (i.e., Dolphin 3D Surgery module) was used to perform virtual treatment objective evaluation by integrating data from cone beam computer tomography acquisition, intraoral scan, and extraoral photographs. The software allowed a comprehensive evaluation of skeletal, dento-alveolar, and soft-tissue disharmonies, qualitative and quantitative simulation of surgical procedure according to skeletal and aesthetic objectives, and, consequently, the treatment of the malocclusion. Using a specific function of the software, the surgical splint was designed according to the pre-programmed skeletal movements, and subsequently, the physical splint was generated with a three-dimensional (3D) printing technology. Once a proper occlusal decompensation was reached, a Le Fort I osteotomy of the maxilla and a bilateral sagittal surgical osteotomy of the mandible were executed to restore proper skeletal relations. The whole treatment time was 8 months. The orthodontic/orthognathic combined treatment allowed to correct the skeletal and the dental imbalance, as well as the improvement of facial aesthetics. Accordingly, the treatment objectives planned in the virtual environment were achieved. Virtual planning offers new possibilities for visualizing the relationship between dental arches and surrounding bone and soft structures in a single virtual 3D model, allowing the specialists to simulate different surgical and orthodontic procedures to achieve the best possible result for the patient and providing an accurate and predictable outcome in the treatment of challenging malocclusions.

## 1. Introduction

Conventional orthognathic surgery plan includes clinical examination and diagnostic tools, such as lateral cephalometric radiographs, plaster models, face-bow, articulators, and photographs [1]. However, traditional planning procedure based on plaster casts neglects the anatomical information of the whole skull, which, in patients with severe deformities, could leave a variation with unpleasant consequences that could lead to significant problems [2].

For these reasons, it would be useful to evaluate not only the occlusion but also the facial soft tissues, jaws, and the underlying skeleton. Three-dimensional (3D) virtual imaging and planning techniques offer the advantage of combining information from the soft tissues of the face, skeleton, and dentition [3]. This technology allows surgeons to design individually appropriate osteotomy plans and orthodontists to evaluate different therapeutic scenarios in a virtual computer environment. In particular, the possibility to integrate 3D models of maxillary and mandibular arches with the reconstruction of the skull form cone-beam computed tomography (CBCT) generates a virtual reliable patient's representation, with realistic reproduction of the skeletal and soft-tissue changes determined by the planned surgery [4].

In the present case report, we present and discuss the orthodontic/orthognathic combined treatment of a skeletal malocclusion correction in a 17-year-old male patient affected by a skeletal class III, facial asymmetry, sagittal and transversal deficiency of the medium third of the skull, dental crowding, and bilateral cross-bite. In particular, the present study will be focused on the effectiveness and advantages of integrating 3D digital technology, from virtual treatment plan to computer-aided design and computer-aided manufacturing (CAD-CAM) systems, in treating complex cases, such as malocclusion requiring orthognathic surgery.

## 2. Materials and Methods

### 2.1. Diagnosis and Etiology

**Figure 1:**
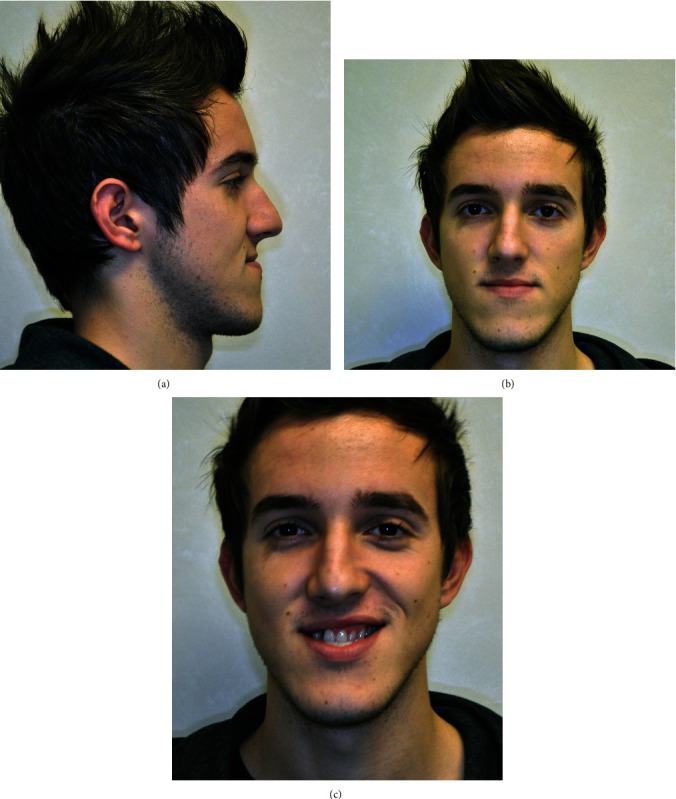
Pretreatment extraoral photographs: (a) right profile view, (b) full face, and (c) smiling.

**Figure 2:**
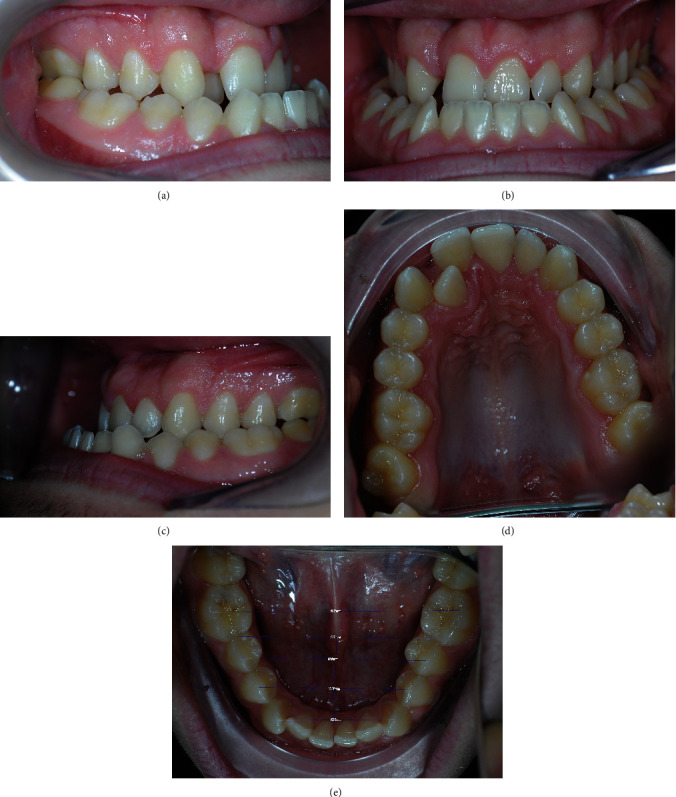
Pretreatment intraoral photographs: (a) right lateral, (b) anterior, (c) left lateral, (d) maxillary occlusal view, and (e) mandibular occlusal view.

A 17-year-old male patient attended an orthodontic consultation to evaluate his craniofacial dysmorphosis and to improve his smile and facial appearance. The extraoral examination showed a severe skeletal class III, a severe lower third facial lengthening with an aging appearance, thin lips with little represented vermillion, and an underdevelopment of the middle third. On a frontal view, the face was asymmetrical with the labial commissure tilted higher from the left side, the chin dislocated to the left, and emimandibular elongation on the right side (Figure 1). The intraoral examination revealed a class III with total reverse bite extending throughout the arch, a transversely reduced maxilla, and a dental crowding in both upper and lower arches. The superior median line was deviated to the right and the inferior median line to the left (Figure 2). No clinically evident signs or symptoms of temporomandibular dysfunction were reported.

**Figure 3:**
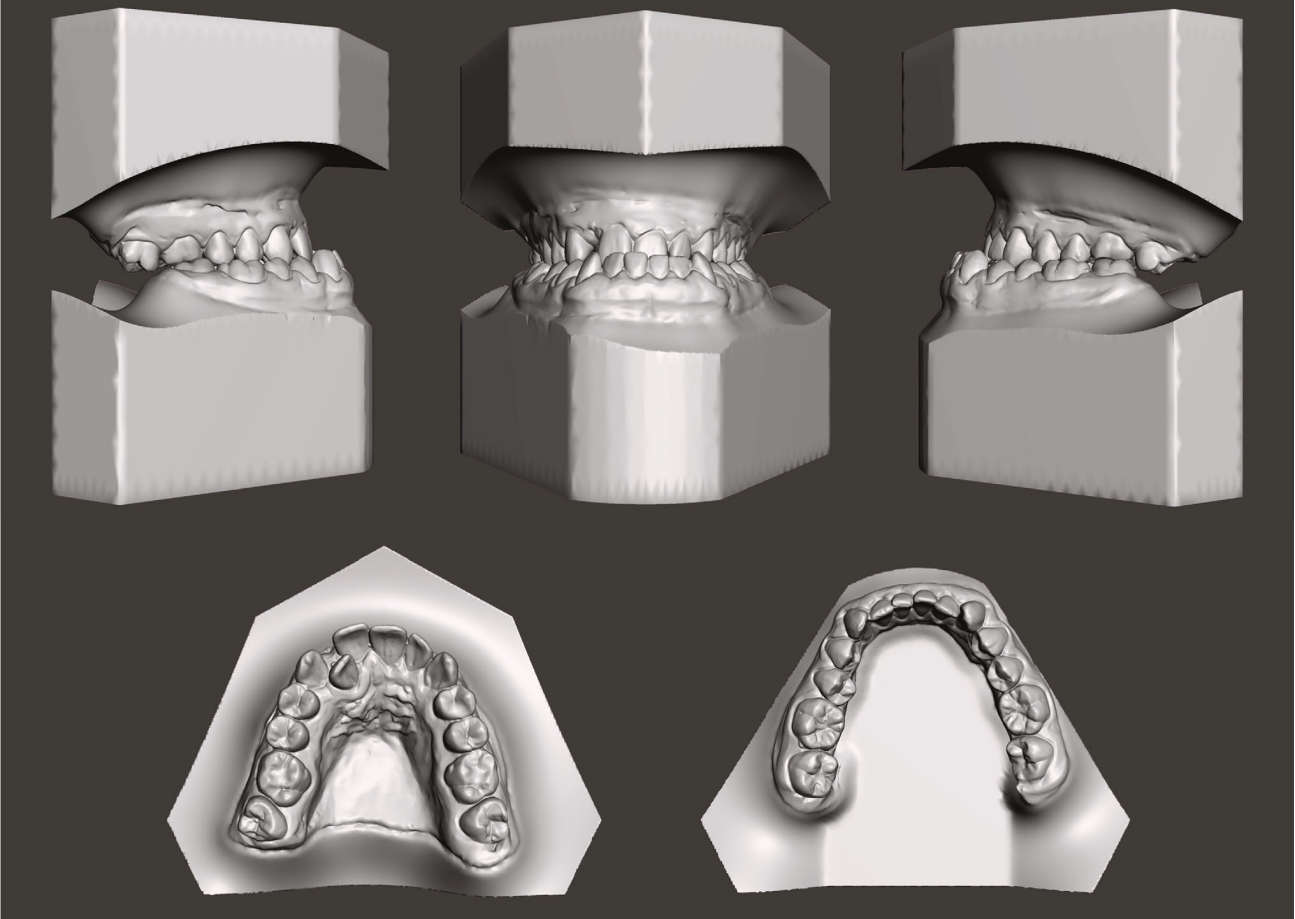
Pretreatment digital dental casts.

The analysis of the 3D models (TRIOS 3Shape A/S, Copenhagen, Denmark) was performed to deeply investigate the occlusal characteristics and the presence of abnormal contacts (Figure 3).

**Figure 4:**
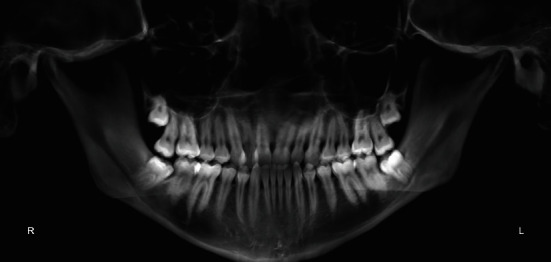
Pretreatment panorex.

Dental panorex showed impaction of both upper and lower third molars (Figure 4).

**Figure 5:**
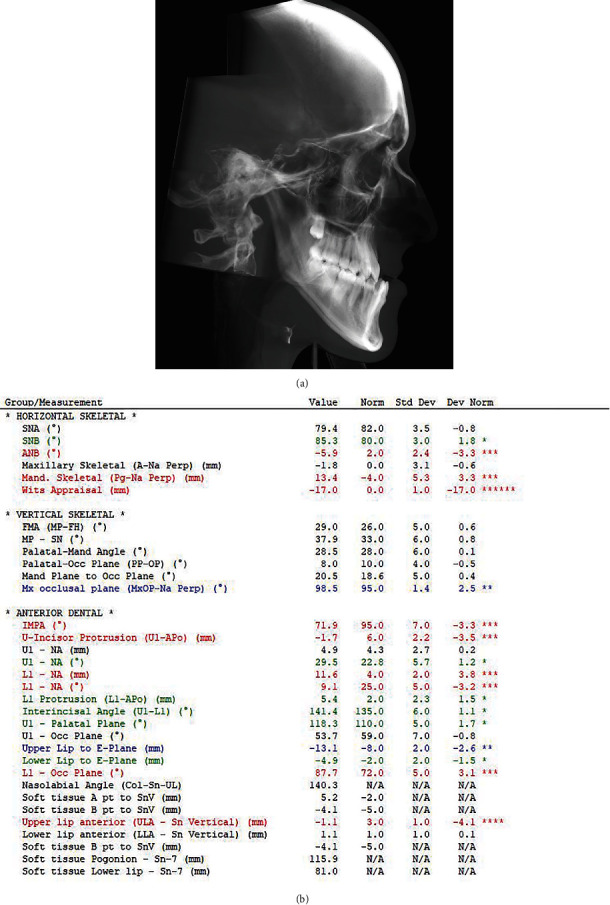
Pretreatment cephalometric analysis (value, norm, standard, deviation, and deviation norm).

The cephalometric analysis confirms a severe class III malocclusion in a hyperdivergent type subject with the following values SNA 79.4°, SNB 85.3°, ANB −5.9°, WITS −17, FMA (MP-FH) 29°, IMPA 71.9°, MP-SN 37.9. A soft-tissue cephalometric analysis was also performed to investigate the vertical and anterior–posterior (AP) projection of the lips and soft-tissue components, according to Arnett. The analysis revealed a severe soft-tissue imbalance according to the following parameters: *A*′ point −1.6, *B*′ point 2.1, Pog′-Sn 3.9, naso-labial angle (Col-Sn′-ULA) 117.7°, upper lip length (Sn′-ULI)17.5 [5] (Figure 5).

CBCT examination was also performed to investigate the entity of the skeletal disharmony, facial imbalance, the location and extension of the mandibular asymmetry, and a preliminary integrated evaluation of soft-tissue imbalance with skeletal components. The CBCT scans were acquired using Scanora 3Dx (Soredex, Tuusula, Finland) equipment with the following machine settings: fold of view (FOV) 130 mm × 145 mm, 6.3 mA.

### 2.2. Treatment Goals

The objectives of the treatment were the correction of both maxillary and mandibular crowding, the correction of both sagittal and transverse skeletal dysmorphosis up to the achievement of class I, and obtaining an improvement in smile aesthetics and facial profile.

### 2.3. Treatment Alternatives

The first therapeutic option involved a multibracket orthodontic treatment with 3.4 and 4.4 extraction, space closure, and resolution of the superior dental crowding. However, this treatment would not allow to achieve an improvement in the profile and middle third aesthetic and the reduction of class III skeletal malocclusion as requested by the patient.

The second option included the miniscrew-assisted rapid maxillary expansion for correction of the transverse maxillary deficiency, supported by the application of miniplates in the lower arch to manage class III correction with the usage of inter-maxillary elastics. However, considering the amount of the sagittal discrepancy as well as the patient's age, this approach would have not assured to reach the aesthetic expectations of the patient. The third option involved a combined surgical-orthodontic treatment consisting on a first approach with multibrackets therapy to decompensate the dysmorphosis and a following maxillofacial surgery with LE FORT I and BSSO (bilateral sagittal surgical osteotomy) for the skeletal correction of the dysmorphosis. However, this path would not permit an adequate transverse expansion of the middle third, limiting the treatment of the skeletal imbalance to the sagittal and vertical component.

The fourth and last option involved a surgically assisted rapid palatal expansion (SARPE) to correct the transverse dimension of the upper jaw, followed by a combined orthodontic-surgical treatment with maxillofacial intervention. LE FORT I and BSSO as reported above.

The patient, supported by the parents, decided for the third option, in order to correct the dento-skeletal dysmorphosis, asymmetry, aesthetic appearance, crowding, and asymmetry. Patient signed an appropriate consent form for the treatment option chosen and for the availability of sensitive data.

### 2.4. Virtual Treatment Objectives (VTO)

The virtual planning was performed with the orthodontic software Dolphin 3D Surgery module, according to the objectives defined by the orthodontist and surgeon. A comprehensive iconographic description of the digital workflow for orthognathic surgery performed with the Dolphin 3D Surgery software is fully documented in the Supplementary Materials.

In particular, the virtual skeletal movements were performed according to the following parameters: (1) correction of the midlines; (2) correction of the occlusal plane: downward pitch movement of the maxilla; (3) correction of AP position of the maxilla. In this regard, the software allows to monitor the changes of specific cephalometric parameters, the SNA angle in this case, during the simulation movement; (4) correction of the AP and vertical position of mandibular jaw; (5) correction of the anterior facial proportion; (6) achievement of soft-tissue balance [6, 7].

### 2.5. Treatment Progress

First, the upper and lower third molars were extracted in order to facilitate osteotomic surgical maneuvers, as well as repositioning and osteosynthesis maneuvers [8].

**Figure 6:**
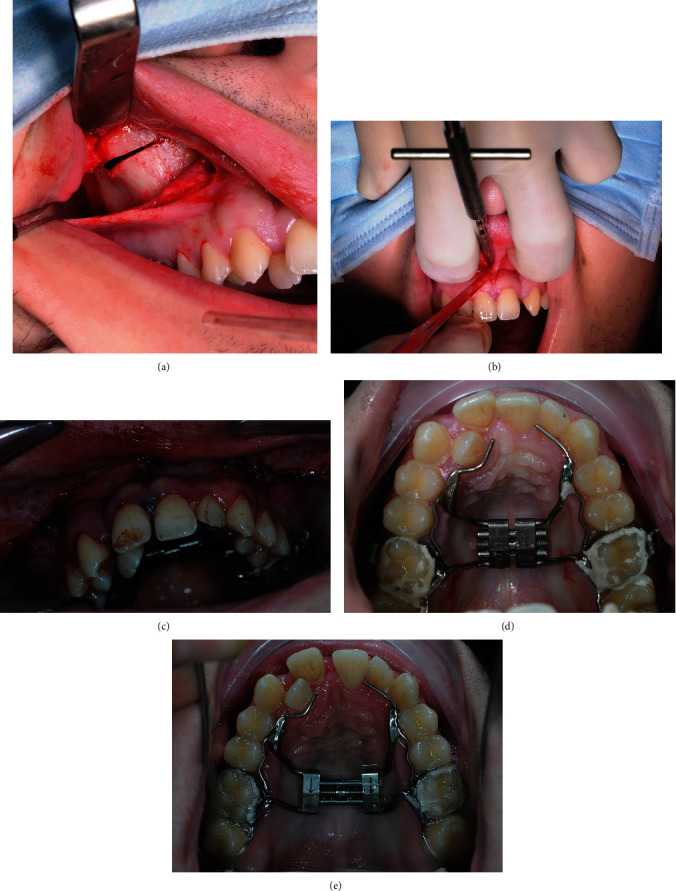
Surgical procedures: (a) osteotomy underneath the zygomatic process bilaterally, (b) osteotomy in the anterior median region, (c) suture, and (d and e) HYRAX maxillary expander pre- and post-activations.

The first phase involves maxillary expansion with SARPE, which consists of a surgical liberation of the sites of resistance combined using orthopedic forces. In this regard, intraoral scans (Trios 3Shape A/S, Copenhagen, Denmark) were sent to an orthodontic digital laboratory for the production of HYRAX maxillary expander, featuring bands on 1.6 and 2.6 and with arms extended to the second molars 1 mm above the gingival margin. The appliance was cemented with glass ionomer cement (KETAC 3M, Saint Paul, MN, USA) 2 days before surgery (Figure 6).

The surgical procedure was performed in the surgery unit of private dental office in Catania; patient underwent conscious intravenous sedation, successfully managed by an experienced anesthetist, in order to reduce intraoperative stress. General anesthesia has been usually advocated for these procedures, with the concern that pterygomaxillary disjunction was too traumatic to be performed under sedation. However, recent evidences have suggested that conscious intravenous sedation is adequate to avoid patient stress experience during SARPE [9].

Infiltration anesthesia (articaine) was administered throughout the upper maxilla at the fornix level; afterward, skeletonization (mucoperiosteal incision and dissection) of the right and left alveolar processes was executed. Osteotomy margins were executed (1) underneath the zygomatic process bilaterally and (2) in the anterior median region, in proximity to the anterior nasal spine, exactly 1 cm above the interproximal contact point between central upper incisors (Figures 6(a)–(c)). A straight dental elevator was used to perform paramedian surgical distraction of the maxilla. The surgical technique used sought to attain a balance between maximum mobilization of the maxilla with a complete liberation of all the buttresses and minimum morbidity to avoid further complications.

Afterwards, 10 activations of the expander screw were administered (Figures 6(d) and 6(e)). Two activations per day were performed until the screw was completely open (10 mm); once the activations were completed, the screw was blocked with passive stainless steel (SS) 0.010 mm. After 6 months of retention, a fixed 0.022 self-ligating appliance with the Damon prescription (Ormco, Orange, CA, USA) was bonded on the buccal surface of all erupted teeth.

Both maxillary and mandibular arches were treated with the same arch-wire sequences, that is, 0.014 Cu 155 NiTi, 0.018 Cu NiTi, 0.014 × 0.025 Cu NiTi, 0.018 × 0.025 NiTi, 0.017 × 0.025 TMA, and 0.019 × 0.025 SS posted. This arch-wire sequence allowed to achieve alignment, leveling, and coordination of the arches, up to pre-surgical preparation with SS rigid arches. In particular, the combination of self-ligating brackets and thermal NiTi arch-wires generates a low-force range biomechanics system, which allow the correction of the dento-alveolar discrepancy without causing periodontal iatrogenic damage [10, 11].

**Figure 7:**
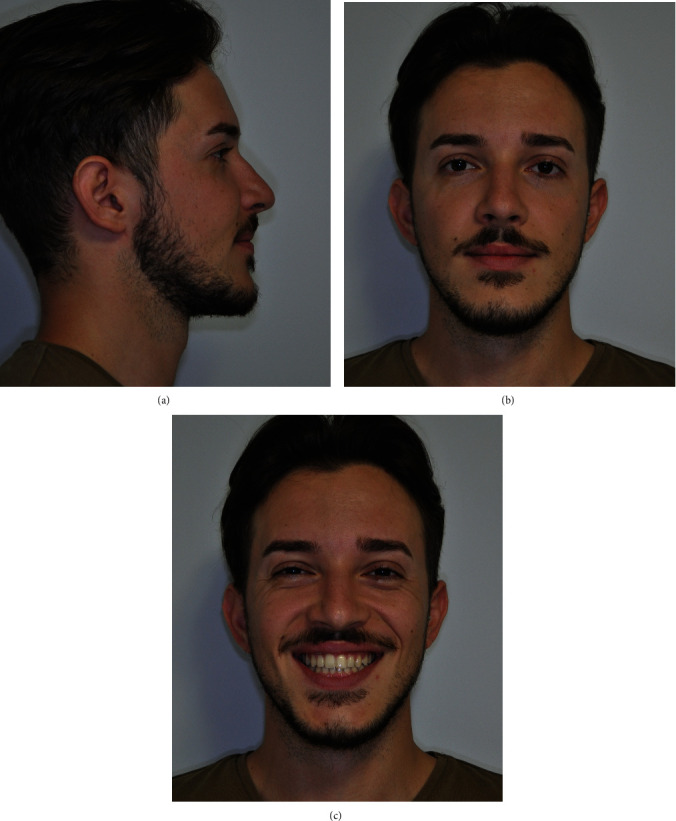
Pre-surgery extraoral photographs: (a) right profile view, (b) full face, and (c) smiling.

**Figure 8:**
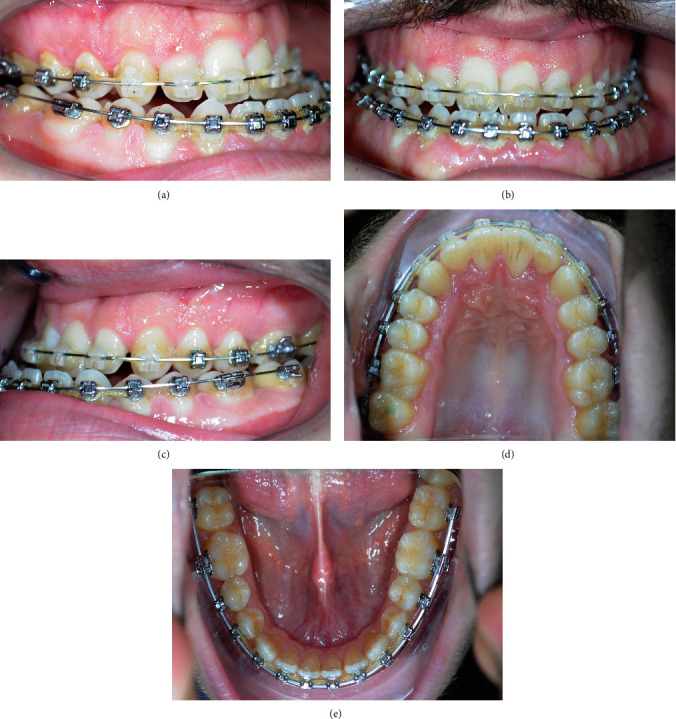
Pre-surgery intraoral photographs: (a) right lateral, (b) anterior, (c) left lateral, (d) maxillary occlusal view, and (e) mandibular occlusal view.

**Figure 9:**
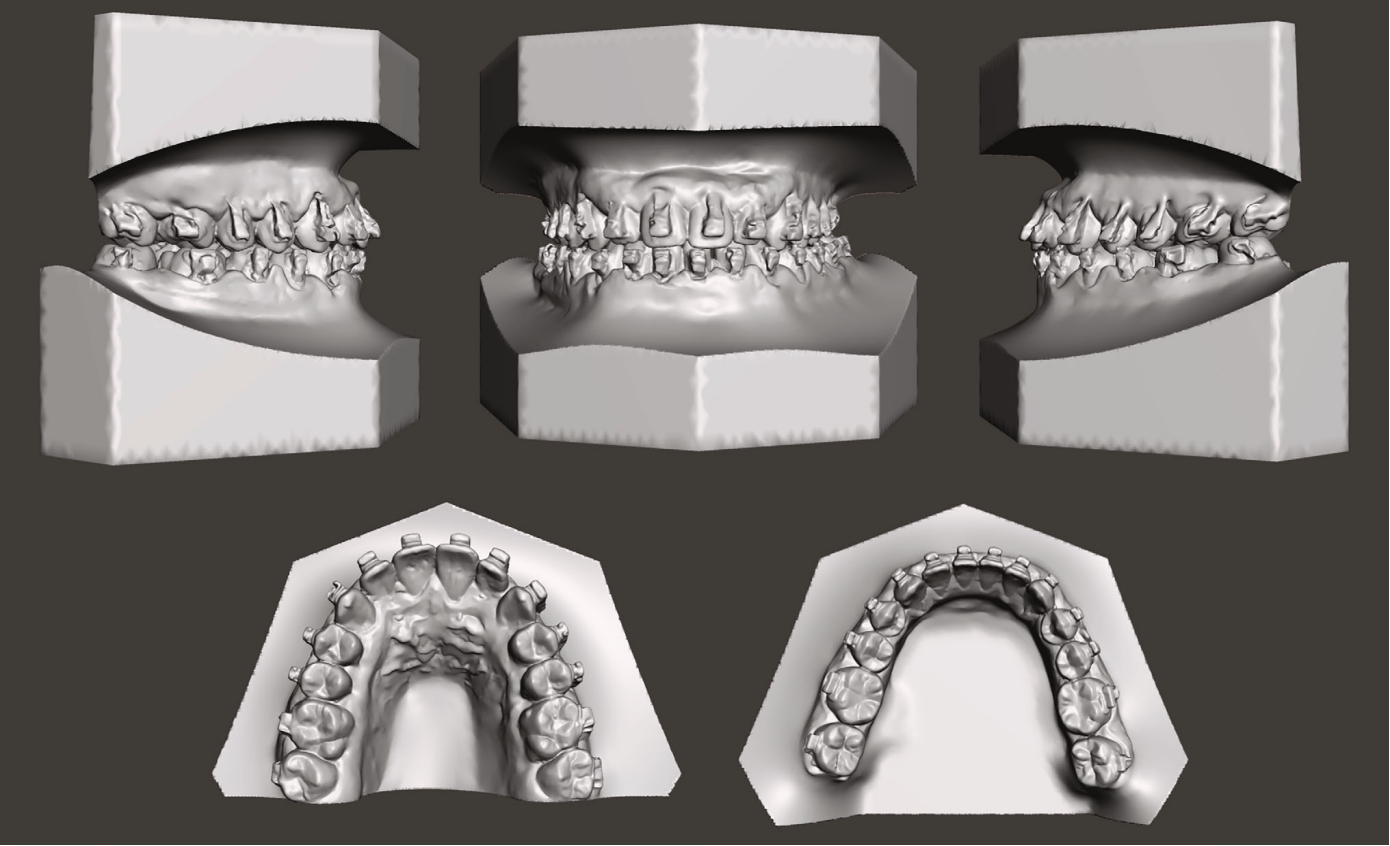
Digital casts oriented according to the planned surgical movements.

Once the arch-wire sequence was completed (with fully transmission of brackets prescription) and dento-alveolar transverse and sagittal compensation eliminated, a pre-surgery diagnostic check-up was performed, including the acquisition of new extraoral and intraoral photographs (Figures 7 and 8; Supplementary Figure 1), intraoral scans (Figure 9), and CBCT scans with large FOV [12]. The intraoral and CBCT scans were imported into Dolphin 3D Surgery module and matched in order to obtain a virtual model of the patient's skull with the dental arches in high definition. Moreover, the software allows to integrate the extraoral patient photographs in frontal view with the surface of the 3D rendered skull model, allowing a comprehensive evaluation of patient skeletal and soft-tissue characteristics before planning virtual surgical jaw movement (Supplementary Figures 2–4).

**Figure 10:**
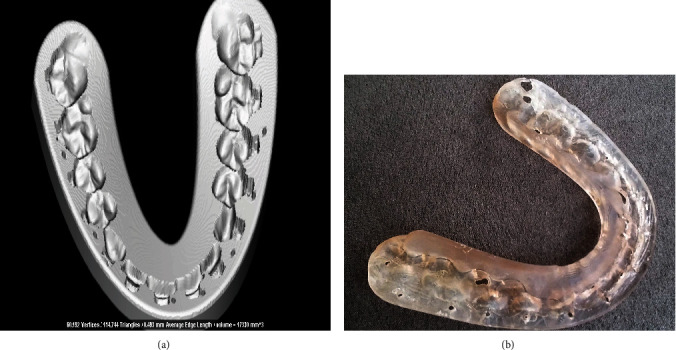
Final splint: (a) STL files and (b) transparent biocompatible resin.

Once the final position of both jaws was defined, the “Splint tool” function of the 3D Dolphin software was used to design a digital intermediate splint and final splints with the insertion of the holes for intermaxillary fixation (Figure 10(a)). In particular, the tool allows selecting the width, thickness, and other parameters of the splint based on your treatment plans. All splint data files were generated in industry-standard STL files∗ to be exported for final prototyping process.

The splints (file .STL) were prototyped with FORMLAB 3D printer (SLA technology, FORMLAB, Somerville, MA, USA) in transparent biocompatible resin (Surgical Guide Resin) with 50 *μ*m of layer thickness (Figure 10(b)). The post-curing process was managed according to the manufacturer's instructions.

The surgical procedure follows the pre-planned settings for LE FORT I and BSSO approaches for the skeletal correction of the dysmorphosis, and finalized with osteosynthesis plates and screws. After the healing of soft tissues, an 8-week home physiotherapy is scheduled to increase speech, chewing, and joint mobility. 10 months after maxillofacial surgery, the case is concluded with rubber bands to increase the intercuspation and finishing arches. The restraint is carried out with upper and lower permanent splint and Essix^®^ appliance during the night.

## 3. Results

**Figure 11:**
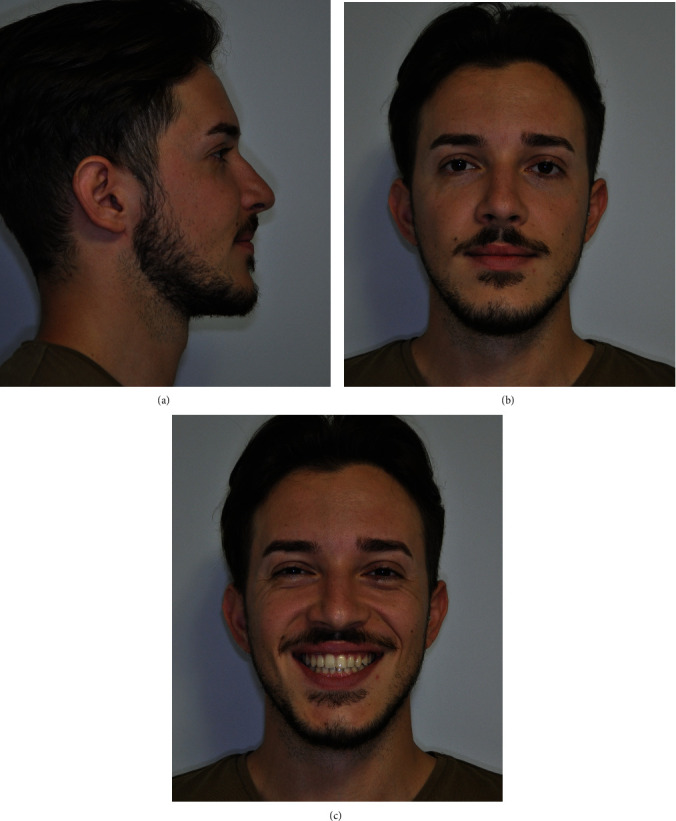
Post-treatment extraoral photographs: (a) right profile view, (b) full face, and (c) smiling.

**Figure 12:**
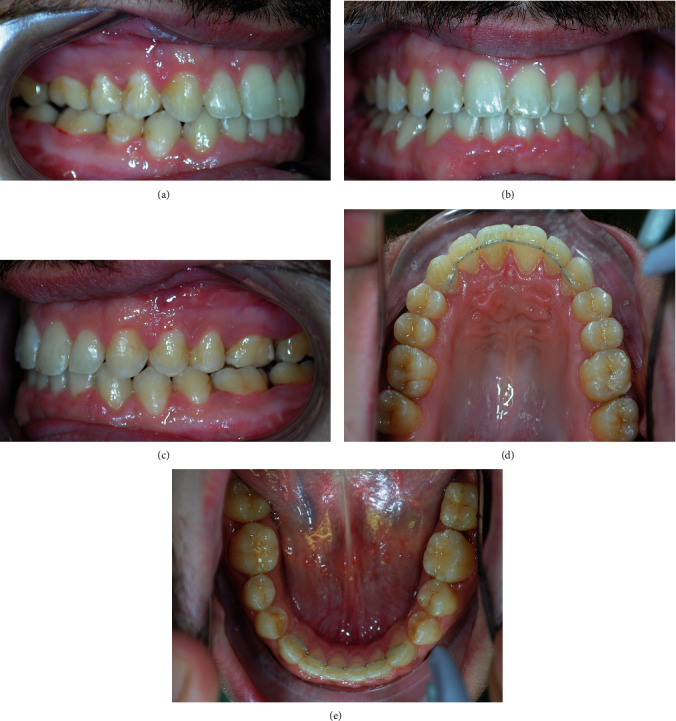
Post-treatment intraoral photographs: (a) right lateral, (b) anterior, (c) left lateral, (d) maxillary occlusal view, and (e) mandibular occlusal view.

**Figure 13:**
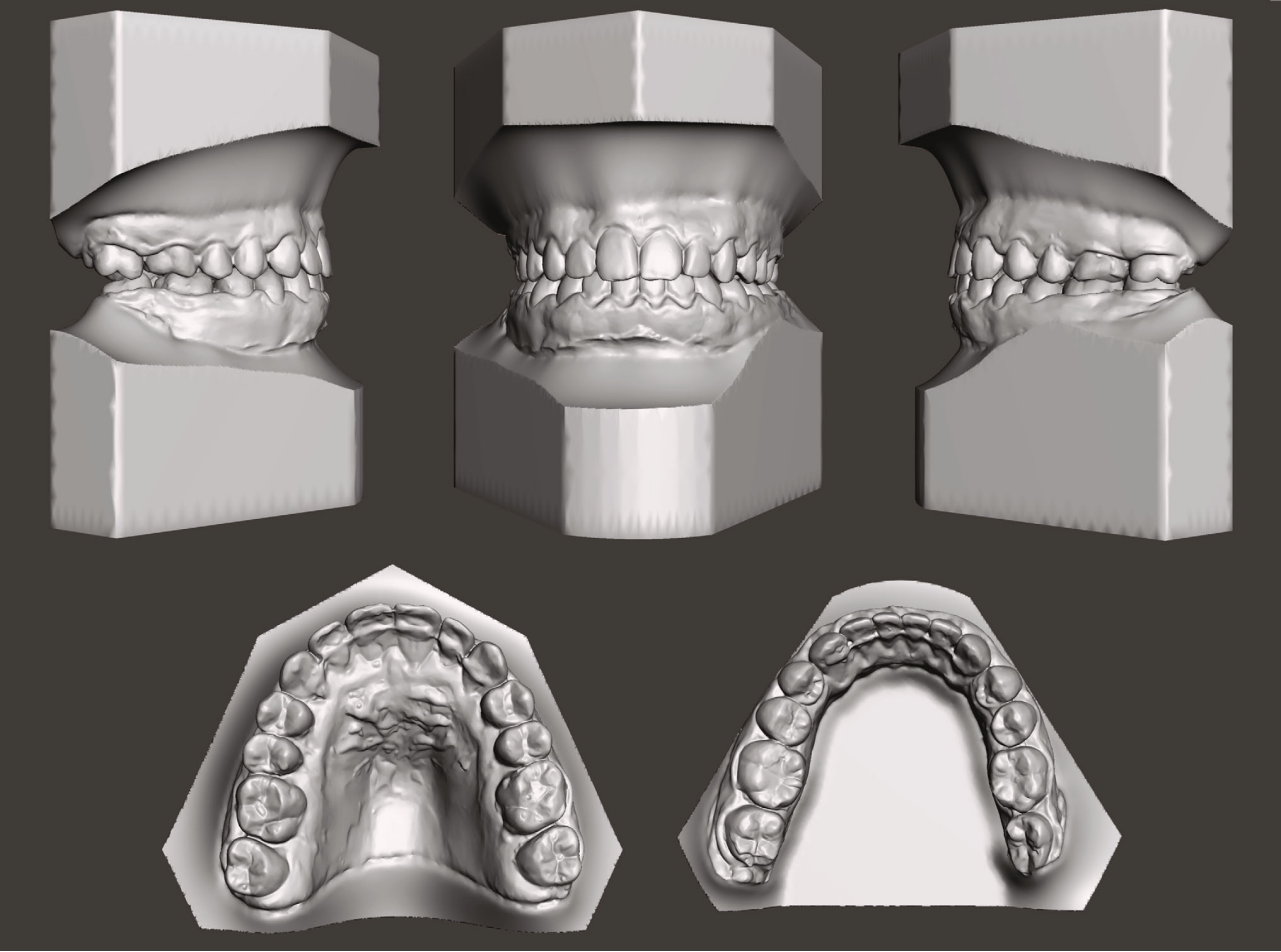
Post-treatment digital casts.

**Figure 14:**
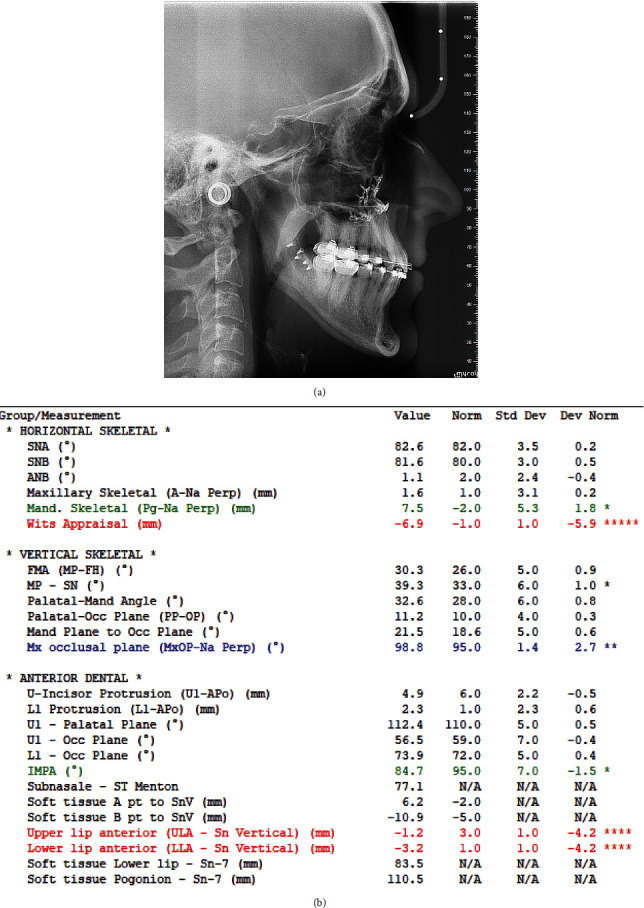
Post-treatment cephalometric analysis (value, norm, standard, deviation, and deviation norm).

**Figure 15:**
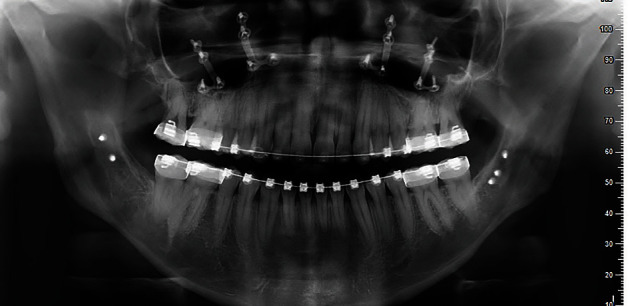
Post-treatment panorex.

**Figure 16:**
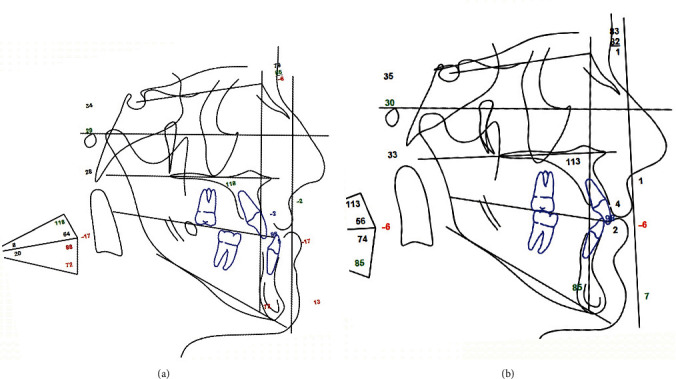
Post-treatment and pre-treatment lateral cephalogram.

**Figure 17:**
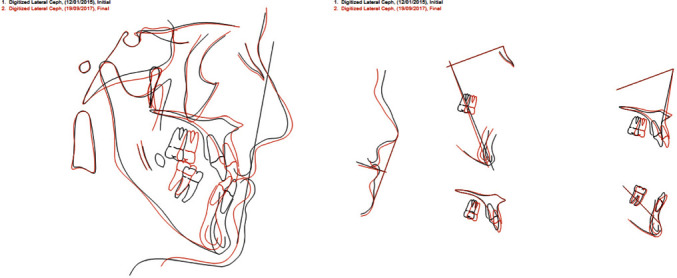
Comparison between post-treatment and pre-treatment lateral cephalogram by anatomical superimposition.

The post-treatment records showed a significant enhancement of both skeletal and dento-alveolar parameters; in particular, facial photographs showed a remarkable improvement in the patient smile and facial aesthetics (Figures 11–15). The comparison between post-treatment and pre-treatment lateral cephalogram revealed that hyperdivergent skeletal pattern (long face) was corrected to normo-divergency on the sagittal plane, with a significant improvement in the ratio between anterior and posterior facial heights, while on the frontal plane, the facial proportions follow the golden proportions of the face (Figure 16, Supplementary Figures 5–7) (Figure 17).

The final cephalometric analysis illustrates the changes achieved by the orthodontic and the surgical treatment. In this regard, it was obtained a correction and normalization of the previous cephalometric values: SNA 83.2° (79.4°), SNB 82.3° (85.3°), ANB 1° (−5.9°), WITS −5.7 (−17), FMA (MP-FH) 30.1° (29°), IMPA 84.5° (71.9°), MP–SN 38.6° (37.9°). The improvement involved not only the skeletal part but also all the aspects related to aesthetics and soft tissues, which now appear very well proportionate in line with the current benchmarks: *A*′ 210 point −2.4 (−1.6), *B*′ point −10.7 (2.1), Pog′-Sn −5.8 (3.9), naso-labial angle (Col-Sn′-ULA) 122° (117.7°°), upper lip length (Sn′-ULI) 20.5 (17.5; Figure 14). The superimposition of pre-treatment and post-treatment cephalograms show the effectiveness of the combination of orthognathic surgery and orthodontic treatment in this patient (Figure 17).

The final orthopantomography shows adequate parallelism and absence of significant root resorption (Figure 15). The final models and intraoral photos show a class I canine with normalization of overjet and overbite, with the achievement of an incisive and canine guide. However, the crowding correction did not predispose to the proinclination of the incisors or periodontal problems (Figures 12 and 13).

The new position of the upper jaw has improved the frontal exposure of the incisal group, restoring muscle tone to the upper lip, which appears well balanced both in frontal and lateral views with a preserved and improved labial competence.

The choice of the combined treatment has achieved all the objectives set, and the patient is satisfied of the aesthetic, functional, and chewing results obtained. The total treatment time was 34 months.

## 4. Discussion

The present case report shows a patient with severe dysmorphosis, in which non-surgical treatment would have not be able to achieve the correction of the dento-facial objectives. Orthognathic surgery allows the correction of skeletal jaws and facial disharmonies and the associated malocclusion, ensuring also aesthetic changes and other benefits, such as chewing, breathing, and speech improvement.

The first treatment phase involved the SARPE, according to the diagnosis of maxillary transverse skeletal discrepancy. In this regard, it could be argued that microimplant assisted rapid palatal expansion would have provided skeletal opening of the midpalatal suture with less invasive procedure [13, 14], increasing the skeletal and dento-alveolar maxillary diameters. However, SARPE, even if it is associated with more complex and longer post-operative stage, is the most stable procedure in the long term since it favors an expansion only of the alveolar processes, not affecting other adjacent maxillary sutures [15].

The second treatment phase involved the orthognathic surgery for the correction of sagittal/vertical skeletal malocclusion, and we referred to digital technology and 3D imaging techniques for preliminary evaluation of the surgical movements of the jaws. In this regard, virtual planning offers new possibilities to visualize the relationship between dental arches and surrounding bone structures in a single virtual model. This approach offers several advantages over conventional planning, including a diagnostic evaluation performed on a 3D virtual model [3]. Virtual 3D planning allows the surgeon and orthodontist to simulate different surgical procedures to obtain the best possible result for the patient, taking into account both skeletal and soft-tissue components, which is fundamental for predicting and achieving adequate aesthetic results [4]. 3D virtual planning facilitates the assessment and correction of the centric relationship in the temporomandibular joint, limiting pre-contacts and post-surgical dislocations; virtual 3D planning is a valuable educational information tool for both the patient and the clinical area [16–18].

For the present case report, we used the Dolphin 3D Surgery software to perform virtual treatment planning. In this regard, the virtual positioning method of the jaws for surgery determines intraoperative success, especially in the prediction of displacement, compared with classic VTO systems and management of gypsum models for several reasons. In fact, this tool permits both planning and presentation of the case showing the patient's skeletal and facial changes in real time and providing a precise surgical guidance [19].

Comparing the photo of the 3D simulation with the final result obtained after the orthodontic-surgical treatment, a significant correspondence of the lateral profile is evident. This is an example of how virtual treatment planning can be reliable in a clinical setting; future studies are warmly encouraged to comparatively assess the planned and post-treatment craniometric and photographic parameters as well as the 3D radiographic parameters.

In computer-aided surgical simulation systems, the virtual occlusal plane is transferred to the patient using surgical splints, which can be fabricated directly using CAD-CAM techniques [20]. In this case, we used 3D printed surgical splints since prototyping technology has been found to be accurate in producing occlusal devices [21–24]. One of the main advantages of prototyped splints is the possibility to select the width, thickness, and other parameters of the splint based on the treatment plans. Afterward, an intermediate splint can be created for the positioning of the maxilla or mandible, according to the order of surgery preferred, and finally, a final splint is generated.

All splint data files are generated in dental and non-dental industry standard .STL files, in order to be produced by the printing process with certified and biocompatible resins. The possibility to directly prototyping surgical splints streamlines the entire digital workflow as well as assures comfortable and accurate occlusal appliance.

## 5. Conclusions

The orthodontic/orthognathic combined treatment allowed to correct the skeletal and the dental imbalance, as well as the improvement of facial aesthetics. Virtual planning allows visualizing the relationship between dental arches and surrounding bone and soft tissues in a single virtual 3D model; thus, the specialists can simulate different surgical and orthodontic maneuvers to achieve predictable outcomes in the treatment of challenging malocclusions.

## Data Availability

The data used to support the findings of this study are available from the corresponding author upon request.

## Abbreviations

SARPE: Surgically assisted rapid palatal expansion
VTO: Virtual treatment objectives
CBCT: Cone beam computer tomography
BSSO: Bilateral sagittal split osteotomy
3D: Three-dimensional
CAD-CAM: Computer-aided drafting-computer-aided manufacturing
FOV: Field of view
AP: Anterior–posterior
SS: Stainless steel
MARPE: Microimplant assisted rapid palatal expansion.

## Supplementary Materials

Supplementary Materials Supplementary Figure 1. On the 3D Surgery module, the first step consists in delimiting the reference areas on the virtual OPT: right and left maxilla, right and left body and mandibular branch. Supplementary Figure 2. The software generates the STL file of this anatomical areas that must be cleaned (eliminating the external areas) in order to have an anatomical section of the part that have to be moved. Supplementary Figure 3. Mandibular and maxillary. STL file with teeth in high definition. Supplementary Figure 4. Once the virtual anatomical pieces have been obtained, the desired osteotomy lines are determined: in this case is made a Le Fort I for the maxilla and a BSSO for the mandible. Supplementary Figure 5. At this point, it is necessary to have the software recognize the cephalometric points by assigning skeletal and soft-tissue points by performing the 3D cephalometry, the software producing the traces and it is possible to start managing. Malocclusion. Supplementary Figure 6. Now everything is ready to plan the displacements. In this clinical case, the upper jaw is first moved as it would be done during the surgery; the software in the associated screen shows the real-time result of the movement performed and determines the three-dimensional parameters by recording them, including the variation in the soft tissues. Then the mandibular position is corrected too. This step can be managed manually or with the automatic function PIGGY BACK: the intraoral scan can also be done on the models and acquired in post correction centric occlusion. The software drags the teeth and the mandible according to the previously recorded occlusion. This system seems to be the best for having the most reliable and predictable guidance of case management. Supplementary Figure 7. Digital design (CAD) of prototyped surgical splint, according to the simulated surgical movements.

## References

[1] Chen Z., Mo S., Fan X., You Y., Ye G., Zhou N. (2021). A meta-analysis and systematic review comparing the effectiveness of traditional and virtual surgical planning for orthognathic surgery: based on randomized clinical trials. *Journal of Oral and Maxillofacial Surgery*.

[2] Baan F., van Meggelen E. M., Verhulst A. C., Bruggink R., Xi T., Maal T. J. J. (2021). Virtual occlusion in orthognathic surgery. *International Journal of Oral and Maxillofacial Surgery*.

[3] Alkhayer A., Piffko J., Lippold C., Segatto E. (2020). Accuracy of virtual planning in orthognathic surgery: a systematic review. *Head & Face Medicine*.

[4] Tonin R. H., Iwaki Filho L., Yamashita A. L. (2020). Accuracy of 3D virtual surgical planning for maxillary positioning and orientation in orthognathic surgery. *Orthodontics & Craniofacial Research*.

[5] Arnett G. W., Jelic J. S., Kim J. (1999). Soft tissue cephalometric analysis: diagnosis and treatment planning of dentofacial deformity. *American Journal of Orthodontics and Dentofacial Orthopedics*.

[6] Elnagar M. H., Aronovich S., Kusnoto B. (2020). Digital workflow for combined orthodontics and orthognathic surgery. *Oral and Maxillofacial Surgery Clinics of North America*.

[7] Plooij J. M., Maal T. J., Haers P., Borstlap W. A., Kuijpers-Jagtman A. M., Berge S. J. (2011). Digital three-dimensional image fusion processes for planning and evaluating orthodontics and orthognathic surgery. A systematic review. *International Journal of Oral and Maxillofacial Surgery*.

[8] Steinbacher D. M., Kontaxis K. L. (2016). Does simultaneous third molar extraction increase intraoperative and perioperative complications in orthognathic surgery?. *The Journal of Craniofacial Surgery*.

[9] Hernandez-Alfaro F., Mareque Bueno J., Diaz A., Pagés C. M. (2010). Minimally invasive surgically assisted rapid palatal expansion with limited approach under sedation: a report of 283 consecutive cases. *Journal of Oral and Maxillofacial Surgery*.

[10] Tecco S., Di Iorio D., Cordasco G., Verrocchi I., Festa F. (2007). An in vitro investigation of the influence of self-ligating brackets, low friction ligatures, and archwire on frictional resistance. *European Journal of Orthodontics*.

[11] Cozzani M., Sadri D., Nucci L., Jamilian P., Pirhadirad A. P., Jamilian A. (2020). The effect of Alexander, Gianelly, Roth, and MBT bracket systems on anterior retraction: a 3-dimensional finite element study. *Clinical Oral Investigations*.

[12] Ludlow J. B., Timothy R., Walker C. (2015). Effective dose of dental CBCT-a meta analysis of published data and additional data for nine CBCT units. *Dentomaxillofacial Radiology*.

[13] Carlson C., Sung J., RW M. C., Machado A. W., Moon W. (2016). Microimplant-assisted rapid palatal expansion appliance to orthopedically correct transverse maxillary deficiency in an adult. *American Journal of Orthodontics and Dentofacial Orthopedics*.

[14] Baik H.-S., Kang Y.-G., Choi Y. J. (2020). Miniscrew-assisted rapid palatal expansion: a review of recent reports. *Journal of the World Federation of Orthodontists*.

[15] Carvalho P. H. A., Moura L. B., Trento G. S. (2020). Surgically assisted rapid maxillary expansion: a systematic review of complications. *International Journal of Oral and Maxillofacial Surgery*.

[16] Leonardi R., Aboulazm K., Lo Giudice A. (2021). Evaluation of mandibular changes after rapid maxillary expansion: a CBCT study in youngsters with unilateral posterior crossbite using a sur-face-to-surface matching technique. *Clinical Oral Investigations*.

[17] Lo Giudice A., Ronsivalle V., Spampinato C., Leonardi R. (2021). Fully automatic segmentation of the mandible based on convolutional neural networks (CNNs). *Orthodontics & Craniofacial Research*.

[18] Leonardi R., Muraglie S., Lo Giudice A., Aboulazm K. S., Nucera R. (2020). Evaluation of mandibular symmetry and morphology in adult patients with unilateral posterior crossbite: a CBCT study using a surface-to-surface matching technique. *European Journal of Orthodontics*.

[19] Schneider D., Kammerer P. W., Hennig M., Schon G., Thiem D. G. E., Bschorer R. (2019). Customized virtual surgical planning in bimaxillary orthognathic surgery: a prospective randomized trial. *Clinical Oral Investigations*.

[20] Chin S. J., Wilde F., Neuhaus M., Schramm A., Gellrich N. C., Rana M. (2017). Accuracy of virtual surgical planning of orthognathic surgery with aid of CAD/CAM fabricated surgical splint-a novel 3D analyzing algorithm. *Journal of Cranio-Maxillo-Facial Surgery*.

[21] Lin H.-H., Lonic D., Lo L.-J. (2018). 3D printing in orthognathic surgery—a literature review. *Journal of the Formosan Medical Association*.

[22] d’Apuzzo F., Minervini G., Grassia V., Rotolo R. P., Perillo L., Nucci L. (2021). Mandibular coronoid process hypertrophy: diagnosis and 20-year follow-up with CBCT, MRI and EMG evaluations. *Applied Sciences*.

[23] Lerario F., Roncati M., Gariffo A. (2016). Non-surgical periodontal treatment of peri-implant diseases with the adjunctive use of diode laser: preliminary clinical study. *Laser in Medical Science*.

[24] De Felice M. E., Nucci L., Fiori A., Flores-Mir C., Perillo L., Grassia V. (2020). Accuracy of interproximal enamel reduction during clear aligner treatment. *Progress in Orthodontics*.

