# Supplementary material for: A Full Computerized Workflow for Planning Surgically Assisted Rapid Palatal Expansion and Orthognathic Surgery in a Skeletal Class III Patient
Source: Case Rep Dent. 2022 Oct 19;2022:6413898. doi: 10.1155/2022/6413898 (PMC9605851; doi:10.1155/2022/6413898)
Supplement: Supplementary Materials — Supplementary Figure 1. On the 3D Surgery module, the first step consists in delimiting the reference areas on the virtual OPT: right and left maxilla, right and left body and mandibular branch. Supplementary Figure 2. The software generates the STL file of this anatomical areas that must be cleaned (eliminating the external areas) in order to have an anatomical section of the part that have to be moved. Supplementary Figure 3. Mandibular and maxillary. STL file with teeth in high definition. Supplementary Figure 4. Once the virtual anatomical pieces have been obtained, the desired osteotomy lines are determined: in this case is made a Le Fort I for the maxilla and a BSSO for the mandible. Supplementary Figure 5. At this point, it is necessary to have the software recognize the cephalometric points by assigning skeletal and soft-tissue points by performing the 3D cephalometry, the software producing the traces and it is possible to start managing. Malocclusion. Supplementary Figure 6. Now everything is ready to plan the displacements. In this clinical case, the upper jaw is first moved as it would be done during the surgery; the software in the associated screen shows the real-time result of the movement performed and determines the three-dimensional parameters by recording them, including the variation in the soft tissues. Then the mandibular position is corrected too. This step can be managed manually or with the automatic function PIGGY BACK: the intraoral scan can also be done on the models and acquired in post correction centric occlusion. The software drags the teeth and the mandible according to the previously recorded occlusion. This system seems to be the best for having the most reliable and predictable guidance of case management. Supplementary Figure 7. Digital design (CAD) of prototyped surgical splint, according to the simulated surgical movements. [file 6413898.f1.docx]

**Supplementary Materials:**

**
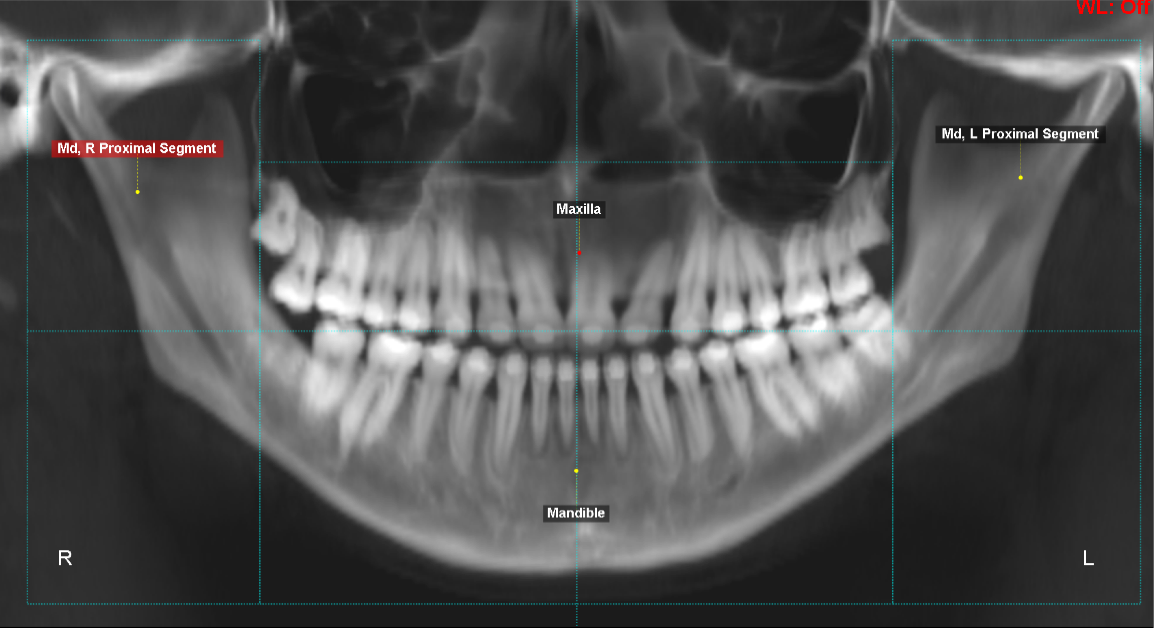
Supplementary figure 1**. On the 3D Surgery module, the first step consists in delimiting the reference areas on the virtual OPT: right and left maxilla, right and left body and mandibular branch.


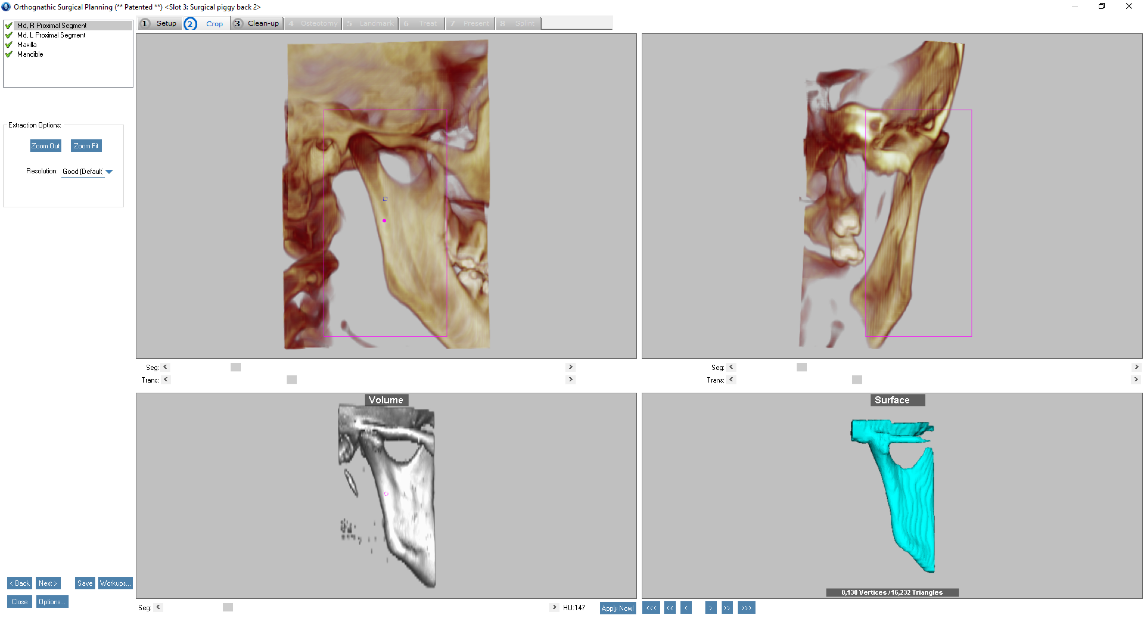
**Supplementary figure 2**. The software generates the .STL file of this anatomical areas that must be cleaned (eliminating the external areas) in order to have an anatomical section of the part that have to be moved.

**
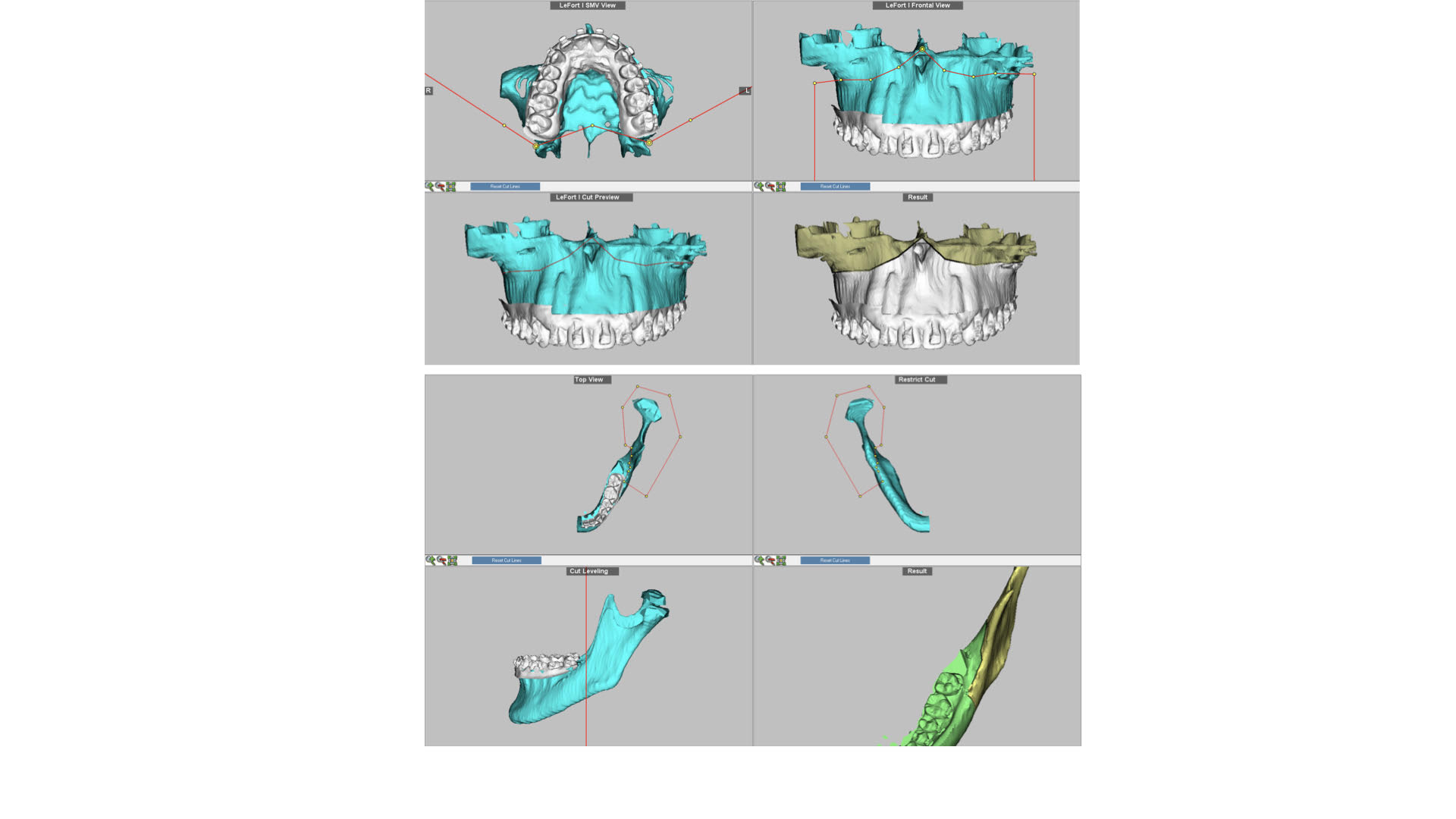

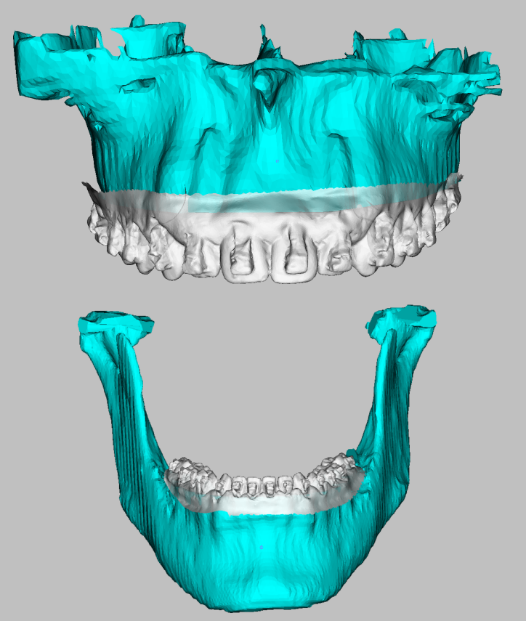
Supplementary figure 3**. Mandibular and maxillary .stl file with teeth in high definition.

**Supplementary figure 4**. Once the virtual anatomical pieces have been obtained, the desired osteotomy lines are determined: in this case is made a Le Fort I for the maxilla and a BSSO for the mandible.

**
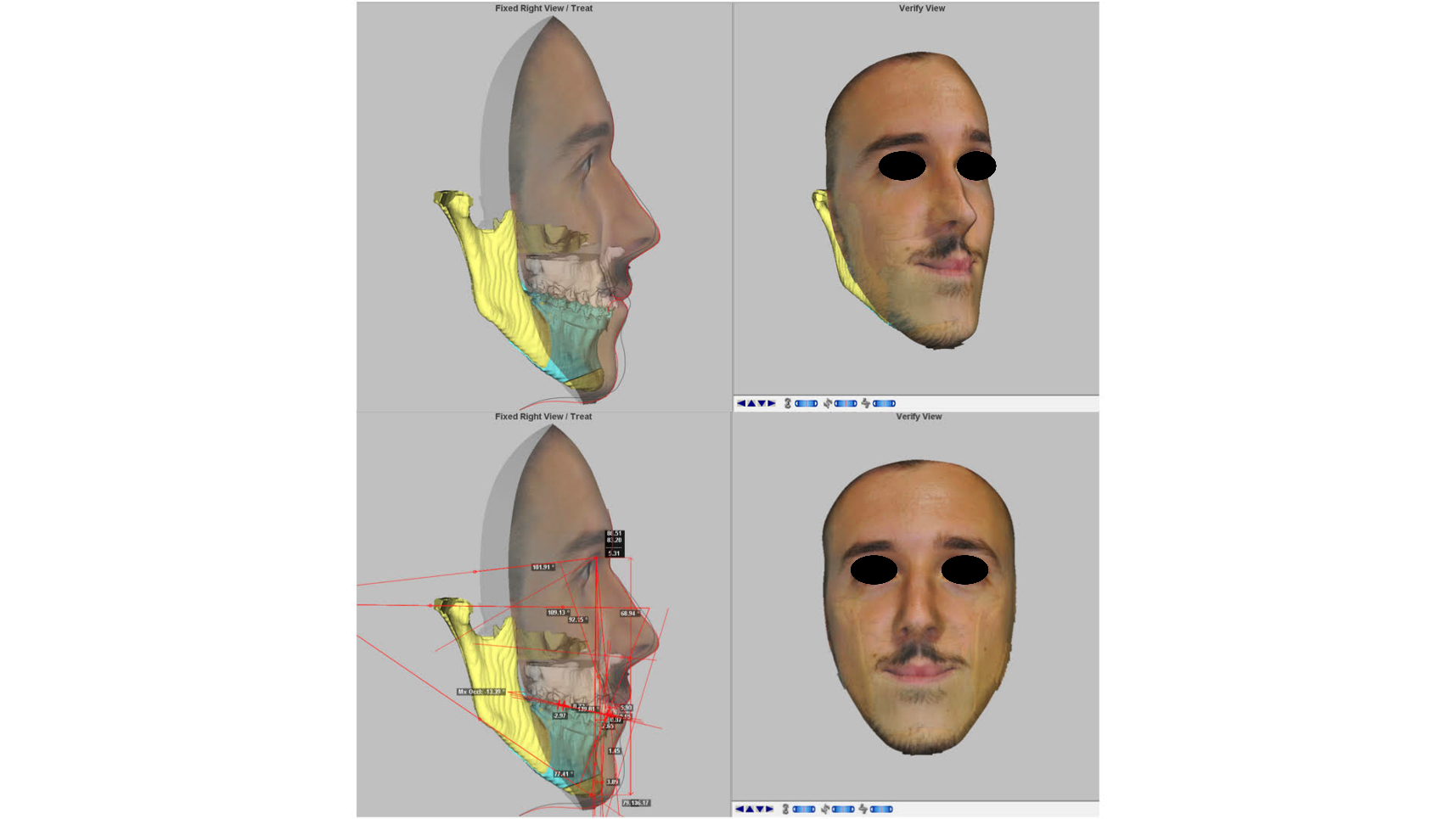

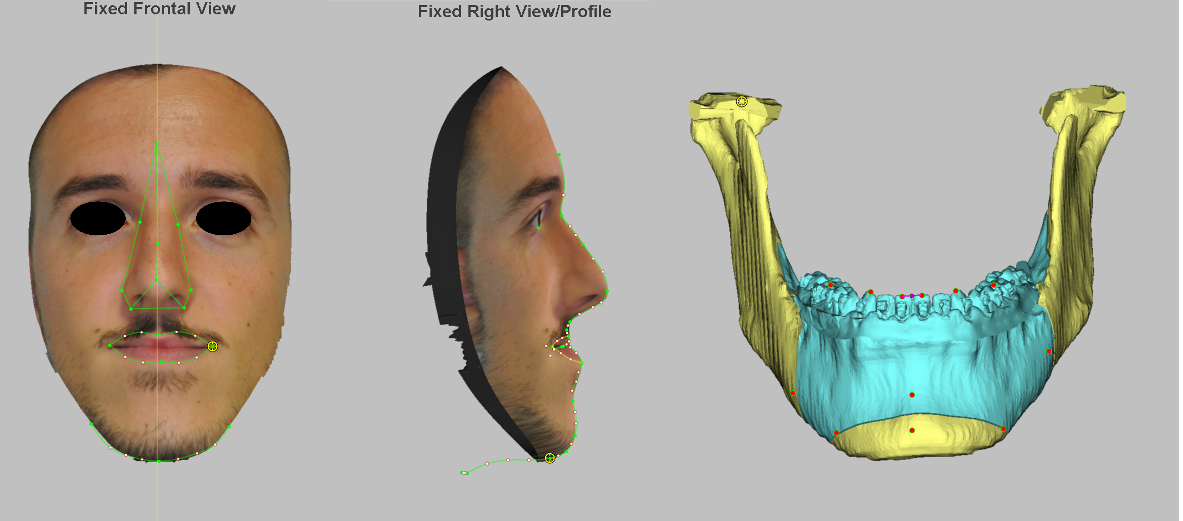
Supplementary figure 5.** At this point it is necessary to have the software recognize the cephalometric points by assigning skeletal and soft tissue points by performing the 3D cephalometry, the software producing the traces and it is possible to start managing the malocclusion.

**Supplementary figure 6.** Now everything is ready to plan the displacements. In this clinical case, the upper jaw is first moved as it would be done during the surgery; the software in the associated screen shows the real-time result of the movement performed and determines the three-dimensional parameters by recording them, including the variation in the soft tissues. Then the mandibular position is corrected too. This step can be managed manually or with the automatic function PIGGY BACK: the intraoral scan can also be done on the models and acquired in post correction centric occlusion. The software drags the teeth and the mandible according to the previously recorded occlusion. This system seems to be the best for having the most reliable and predictable guidance of case management.

**
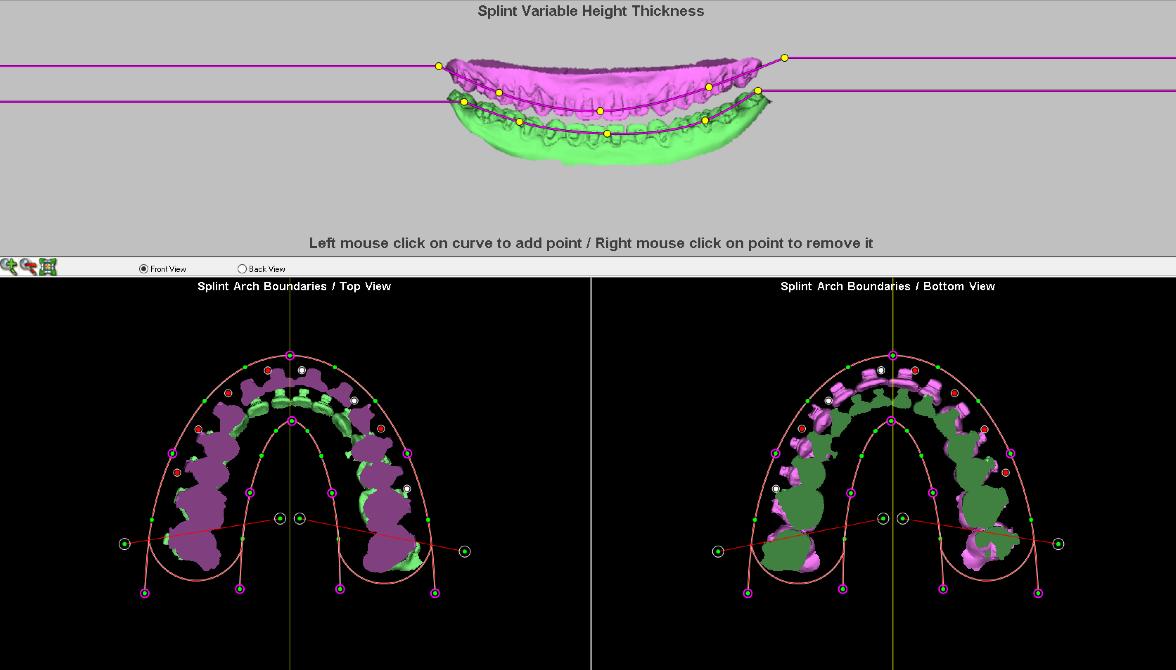
Supplementary figure 7.** Digital design (CAD) of prototyped surgical splint, according to the simulated surgical movements.
